# Monthly Summary

**Published:** 1870-06

**Authors:** 


### MONTHLY SUMMARY.

*Convulsions.*—In these cases, the effect of oiling is sometimes truly surprising, the fit ceasing before the completion of the operation, and not subsequently returning. A patient informs me that whenever she observes the symptoms which used to precede convulsions in her boy, she at once oils him, when a calm sleep follows, from which the child wakes up refreshed.—*Dr. Knaggs. Medical Gazette.*

*Strychnia an Antidote to Chloral.*—Liebreich has recently demonstrated the curious fact that a poisonous dose of strychnia counteracts the effects of chloral, by the following experiments: A subcutaneous injection of two grammes of chloral in a rabbit (about equivalent to 100 grammes for a man of ordinary size) produces speedy collapse and death in about half an hour; an injection of .0015 gramme of strychnia in a second rabbit, causes tetanus and death in about twelve minutes; if to a third rabbit, however the same dose of chloral be administered and followed, when it begins to act, by the same dose of strychnia, the animal soon revives, and in an hour and a half gets on its legs and feeds as usual.—*Medical Gazette.*

*Methylic Ether as an Anæsthetic.*—At the Medical Society of London, on Monday night, Dr. Richardson made a communication on the application of methylic ether as a general anæsthetic, Methylic ether is made by mixing one part of sulphuric acid with two of pure methylic alcohol, and applying the heat. The ether passes over as a gas, having an ethereal odor, and a vapor density of 23, taking hydrogen as unity. To fix the gas, Dr. Richardson passes it slowly through pure ethylic ether, of specific gravity ·730, and boiling point of 95° Fahr.: the gas is being absorbed for several hours, and the result is an ethylic ether saturated with methylic. This is the fluid employed for anæsthesia. Two drachms of the fluid are poured upon domette in a simple mouth-piece, which also covers the nostrils, and the vapor from the surface of the domette is directly inhaled. Dr. Richardson reported eleven cases of tooth extraction in which he had successfully anæsthetised with methylic ether, at the National Dental Hospital; and since monday, Mr. Gregson has used it at the Dental Hospital of London, also with great success. Two peculiarities, at least, may be mentioned, as pertaining to the action of the new carnotic :—(1) That it produces quick relaxation of the muscles; (2) That while the patients under its influence are unconscious of pain, they are capable of performing what appear conscious acts, which acts on recovery are entirely forgotten. The anæsthetic sleep is induced usually within a minute and a half, recovery being perfected as quickly; in no period of the anæsthetic is there asphyxia, and the pulse undergoes little alteratiin. In short, from the experience as yet obtained, there is promise that, for short operations at all events, methylic ether will fill an important place in our list of remedies. The chemical composition of the ether is $(CH_3)$ 20—*Lancet.*

---

*Recipe for Burns.*—Make a saturated solution of alumn (four oz. in a quart of hot water.) Dip a cotton cloth in this solution and apply immediately on the burn. As soon as it becomes hot or dry, replace it by another. and continue doing so as often as the cloth dries, which at first will be in a very few minutes. The pain will immediately cease; and after twenty-four hours of this treatment, the burn will be healed, especially if commenced before blisters are formed. The astringent and drying qualities of the alum will entirely prevent their formation. The deepest burns, such as those caused by boiling water, drops of melted metal, phosphorous, gunpowder, fulminating powder, etc., all have been cured by this specific.—*Chicago Medical Journal.*

---

*Syrup Ether.*—This preparation is much used in France, in cases where the employment of ether is indicated; it will be found especially useful in sick headache. The following formula furnishes a syrup rich in the medicinal principle, of a fixed composition, and which is not disturbed by the variations of temperature: Introduce into a flask 44 grains of sugar; 49 grains of distilled water; 5 grains of alcohol, 90°: and 2 grains of pure ether. Agitate and preserve.—*Eclectic Medical Journal.*

---

*Lactic Acid in Croup.*—℞. Acid lactici gtt. xv ad gtt.xx; aq. ℥ ss M., may be used to advantage in croup. This quantity to be used every half hour with an inhaling apparatus, diminishing the acid to gtt. x, then to gtt. v, and inhaling it but every half hour for two hours, when the dyspnœa decreases, which is said to disappear entirely in seven, ten or twelve hours. Protect the eyes and face from the vapor. Dr. Webb prescribes, beside, this local treatment, the following internally: ℞. Sodæ bicarb.s ʒ ij; aq. ℥ iv. M. Sig. A tablespoonful every half hour, or every hour, till the dyspnœa disappears.—*Medical Gazette.*

---

*Valuable Combination.*—A correspondent, Dr. C. W. Davis, of Iowa, writes; "Sulphate of zinc and chlorate of potass. *intimately ground* together in equal quantities will be found a most satisfactory remedy. The various forms of stomatitis, sore throat, ophthalmic, and many cntaneous diseases, yield at ouce, by its use. I was led to make this combination to economize space in my vial case. Instead ef carrying both the zinc and potash, I carry the combination. Even in internal administration the zinc adds to the efficiency of the chlorate.

Ten grains to the oz. of water as a collyrium. 1 drachm to 4 oz of water as a wash in stomatitis, and gargle in sore throat, to be used stronger if necessary as a lotion. The zinc and potash must be *most* intimately ground together to an impalpable powder to insure its perfect and certain efficacy. From the efficiency and curative power of this combination there is evidently a *physiological* affinity."—*Medical and Surgical Reporter.*

---

*Ergot to Arrest Excessive Perspiration.*—Dr. Christman, it is stated, in the *Wurtemb. Med. Corresp. Blatt*, 1869, No. 20, has seen in many cases of excessive perspiration, a dimunition of the cutaneous discharge ensue promptly upon the exhibition of ergot (8 to 10 grammes four times a day)—*Centralblatt f. d. Med. Wissenschaften.*—*American Journal of Medical Sciences.*

*Charcoal.*—Carbon in proportion to its porosity, possesses the power of absorbing many gases in an equal degree. It takes up but little hydrogen, much more oxygen, a large quantity of sulphuretted hydrogen, and a still greater quantity of ammonia. Hence charcoal is correctly used to remove bad smells from rooms, or to prevent the air in them from being contaminated by the effluvia from offensive diarrhœas, dysenteries, foul ulcers, etc.

To effect the first purpose, small doses of charcoal should be given internally several times a day, and some should be put in [illegible]

Charcoal also [illegible] many solutions various coloring matters, and some bitter substances, alkaloids, and mineral matters. Hence Dr. Garrod has recommended it in poisoning by corrosive sublimate, arsenic, morphia, stychnine, belladonna, &c. It also precipitates the coloring matter of urine, and at the same time carries down all the uric acid, and some of the urea, but none of the sugar which is contained in diabetic urine. For these purposes animal charcoal is the best.

It is given with very great success in certain diseases of the stomach, in which its good effects cannot rationally be ascribed to a mere mechanical influence. Thus, it is said to ease the pain of many painful gastric affections, such as chronic ulcer, and neuralgia, and also to check heartburn. But its action is most pronounced in flatulence which it very greatly lessens, and also removes the extreme depression of spirits which accompanies it. It also appears to check the flatulence which arises from the irregular fermentation of food, and the hysteric form which may not be connected in any way with changes in the food.

Most of the charcoal which is taken passes out of the body with the fæces, but a little is said to find its way into the blood, lymphatics, and glands.

It may be given mixed with equal quanities of magnesia bismuth. —*Medical Gazette.*

---

*Amputation Extraordinary.*—A French lecturer on surgery once concluded his discourse on amputations by remarking that the connexions of the head to the trunk are so numerous and so important that the amputation of that member could hardly be undertaken with any reasonable prospect of success. The snbjoined paragraph, which we clip from the Cincinnati *Enquirer*, would seem, however, to show that he was entirely in error:—

"The following extraordinary narrative comes to Chili from San Juan, Argentine Republic: There existed in San Juan an individual eighteen years of age, who had two heads, both of them provided with a countenance at once expressive and of regular outline, each looking toward the sides of the owner, and being united by the posterior part of the cranium. One of the scientific European commissioners, at present traveling through the Argentine Republic proposed to operate upon the individual, taking away one of the heads. The proposal was accepted, and the operation successfully performed on the 13th ult., without causing the least pain to the patient notwithstanding that chloroform was not administered and the operation lasted six minutes. The operation, and the subsequent cure of the parts, is presented as being something marvelous. The head has been preserved in spirits, and will shortly be exhibited in this country."

South America appears to be the surgical Eldorado. It will be remembered that from thence emanated the story of "union by the first intention" effected by a new process between the body of the decapitated convict and the head of his companion in misfortune.—*Medical Gazette.*

---

*Uses of Carbolic Acid.*—I have used carbolic acid in several different cases with much success, and therefore desire to call attention to it.

My first case was one of the sore mouth peculiar to nursing women. When first seen it was of long standing. There appeared to be no sound flesh in the mouth, and large pieces were daily sloughing out. I at once commenced the application of the acid, and in two days the sloughing ceased, and the fetid breath was much diminished. In two weeks the mouth was well. Once during the treatment, the acid was left off, and other washes used for twenty four hours, when the patient began to suffer much from dryness, heat, and pain in the mouth. The acid was again used and relief was almost immediate.—*Med. News.*

---

*Richardson's Painless Knife.*—At the meeting of the British Medical Association, " Dr. B. W. Richardson read a 'Note on a new method of Painless Cutting in Surgery.' The author placed before the section a knife consisting of a revolving blade, and which divided with such rapidity that superficial incisions could be made with it without pain. The revolutions were about twenty-five per second, but the speed might be increased. The knife in its action illustrated that an appreciable interval of time is necessary for fixing an impression on the mind, and for the developement of consciousness. He hoped he would soon be able to give to the surgeon a small pocket instrument with which to open abscesses, and perform many minor operations painlessly, without having recourse to either general or local anæsthesia."—*Medical Gazette.*

---

*To Remove Foreign Bodies from the Ear.*—The least painful and most effective means is to envelop the extremity of a probe with adhesive plaster, and, after warming it, apply it directly to the foreign body, allowing it to remain in contact until cooled, when it may be withdrawn easily and painlessly.—*Oregon Medical & Surgical Reporter.*

---

*Oxalic Acid for the Sting of a Bee.*—J. Weaver of Kingstown, Ind., from personal observation, recommends a topical application of a strong solution of oxalic acid to relieve the pain and discuss the inflammation produced by the sting of a bee. He mentions several cases where his topic has given almost instant relief.—*Journal Materia Medica.*

---

